# Biopolymer Micro/Nanogel Particles as Smart Drug Delivery and Theranostic Systems

**DOI:** 10.3390/pharmaceutics15082060

**Published:** 2023-07-31

**Authors:** Susana C. M. Fernandes, Garbine Aguirre

**Affiliations:** 1 CNRS, IPREM-UMR 5254, Universite de Pau et des Pays de l’Adour, E2S UPPA, 64000 Pau, France; 2 MANTA—Marine Materials Research Group, Universite de Pau et des Pays de l’Adour, E2S UPPA, 64600 Anglet, France; 3 Bio-Inspired Materials Group: Functionalities & Self-Assembly, Universite de Pau et des Pays de l’Adour, E2S UPPA, 64000 Pau, France

## 1. Introduction

In recent years, micro/nanogels have become an important topic of interdisciplinary research, especially in the fields of polymer chemistry and material science, with a focus on their use in drug delivery applications. In this regard, micro/nanogels can potentially revolutionize conventional therapy methods thanks to their incomparable chemical and physical versatility. These stem from the unique combination of their simple synthesis, large surface area, variation of their volume in response to different stimuli, and their ability to contain different types of therapeutic agents in a single system [1].

In general, biocompatibility, and even biodegradability, is required for the above-mentioned medical applications [2,3]. In this sense, the use of biopolymers is considered an appropriate alternative to solve both issues. The diverse compositions, tunable physical behavior, and wide variety from which to choose offer the possibility of developing a huge variety of biopolymer micro/nanogels suitable for different eco-friendly applications [4]

This Special Issue of *Pharmaceutics* highlights advances in the use of biopolymer-based nano/microgels to encapsulate molecules with different therapeutic applications together with new synthesis and/or functionalization approaches to optimize their properties as delivery systems.

Of the papers presented in this Special Issue, four papers study delivery applications [5,6,7,8], four papers focus on optimization by functionalization for delivery applications and/or new synthesis approaches [9,10,11,12], and three review articles evaluate the design and potential applications of micro/nanogels [13,14,15].

From the delivery point of view, Suner et al. developed micro/nanoparticles derived from chondroitin sulfate, which is a polymer native to the eye, for the treatment of bacterial ulcers on the cornea [6]. The biomaterials proposed presented higher and controllable antibiotic loading ability together with lower toxicity, with sustainable drug release kinetics being promising candidates to replace currently used systems as drops and pills. Suneetha and coworkers investigated the combination of chemo and photothermal cancer therapy using doxorubicin-loaded fungal-carboxymethyl chitosan functionalized polydopamine (Dox@FCPDA) nanoparticles [5]. Newly developed Dox@FCPDA nanoparticles exhibited pH-responsive drug release properties as well as enhanced cytoxicity towards cancer cells, demonstrating their potential as photothermal chemotherapy vehicles to treat cancer.

Micro/nanogel particles, due to their sponge’s structure, present an extraordinary ability to contain not only small therapeutic molecules but also macromolecules. In this regard, the publication of Hinkelmann et al. aimed to study the use of stem cells instead of whole-tissue fragments for bone tissue engineering [7]. For that, siRNA was loaded to gelatin microparticles cross-linked with an anhydride-containing oligomer, and then, loaded-microparticles were aggregated with human mesenchymal stem cells. Those microtissues were able to mimic bond autografts in bone defects. The publication of Schötz and coworkers reported the potential of polyglycerol-based redox-responsive nanogels as nanocarriers system for therapeutic proteins delivery [8]. This research demonstrated the reproducible synthesis of monodisperse nanogels with high encapsulation efficiencies of cytochrome C protein and its sustained release by redox degradation of nanogels. In addition, the low cytotoxicity data obtained together with the observed cell internalization ability of the nanogels demonstrated their applicability as protein delivery platforms.

Functionalization of the surface of micro/nanogels is an effective approach for targeted treatments. In the study of Fuster et al., the surface functionalization of silk nanoparticles with folic acid was reported [9]. Functionalized nanoparticles exhibited improved cellular uptake folate-receptor mediated and thus improved antitumor activity of ibrutinib anticancer drugs. Al-Obaidy and coworkers reported the cationic functionalization of shellac nanoparticles to boost the antimicrobial action of chlorhexidine drug [12]. The results showed an enhancement of the antimicrobial effect of functionalized nanoparticles due to their higher accumulation on the microbial cell walls allowing the release of the drug close to the cell membrane. The publication of Lai et al. proposed an elegant gelatin methacryloyl-based microgel synthesis approach using a microfluidic method [11]. Monodisperse microgels exhibited tunable properties (size and degree of swelling) and loading of bioactive agents using flow-focusing microfluidic geometry and varying various fabrication conditions. Gugleva et al. reported the development of a hybrid drug delivery platform for intravesical administration to achieve maximum therapeutic efficacy [10]. The hybrid drug delivery system was based on the curcumin and gentamicin sulfate model drugs simultaneously loaded niosomes, i.e., vesicular carrier, into thermosensitive in situ gel. The hybrid system presented promising antibacterial activity and sustained release patterns, and is thus considered a suitable platform for intravesical delivery.

The three reviews published in this Special Issue address the use of different biopolymer-based micro/nanogels for biomedical applications as drug carriers [13,14,15]. Altuntaş and coworkers have published a very interesting overview of the use of different biopolymers in the design of nanogels together with their most recent advancements in applications for the delivery of drugs and biomolecules [13]. In the study of Froelich et al., micro/nanogel fabrication methods using natural gums and their derivatives together with their application as drug carriers are highlighted [14]. In the review by Marsili et al., dual and multi-responsive chitosan-grafted-poly(N-vinylcaprolactam) microgels synthesized by ionotropic gelation are shown for oncological applications [15]. 

In general, in this Special Issue, original articles and literature reviews are disclosed that allow for understanding the extraordinary properties of biopolymer-based micro/nanogels for controlled delivery applications and giving future perspective of these biomaterials.
